# Association of Genetic Polymorphisms of Fibrinogen, Factor XIII A-Subunit and α_2_-Antiplasmin with Fibrinogen Levels in Pregnant Women

**DOI:** 10.3390/life11121340

**Published:** 2021-12-03

**Authors:** Christian Schwedler, Guido Heymann, Larisa Bukreeva, Berthold Hoppe

**Affiliations:** 1 Institute of Laboratory Medicine and Pathobiochemistry, Charité—Universitätsmedizin Berlin, 13353 Berlin, Germany; christian.schwedler@charite.de; 2 Institute of Laboratory Medicine, Unfallkrankenhaus Berlin, 12683 Berlin, Germany; guido.heymann@ukb.de; 3 Medicover Berlin-Mitte MVZ, 10117 Berlin, Germany; larisa.bukreeva@medicover.de

**Keywords:** fibrinogen, factor XIII, α_2_-antiplasmin, genotype, fibrin crosslinking, inflammation, pregnancy

## Abstract

Fibrinogen synthesis is stimulated by proinflammatory triggers and depends on α-, β- and γ-fibrinogen (*FGA*, *FGB*, *FGG*) genotypes. Constellations of fibrinogen, factor XIII A-subunit (*F13A*) and α_2_-antiplasmin (*A2AP*) genotypes predisposing for dense fibrin gels with high antifibrinolytic capacity (e.g., *FGB* rs1800790 A-allele carriage in *F13A* 34Val/Val or *A2AP* 6Arg/Arg wildtypes) are related with reduced inflammation. As both relationships are likely to influence each other, we tested whether the association of fibrinogen genotypes with fibrinogen levels is influenced by *F13A* and *A2AP* genotypes in a population under proinflammatory stress. In total, 639 women were followed during pregnancy (2218 observations). The relationship between fibrinogen genotypes and levels was statistically assessed in univariate and multivariate analyses without and with stratification for *F13A* Val34Leu and *A2AP* Arg6Trp. Strong associations with fibrinogen levels could be found for *FGB* rs1800790G > A, *FGA* rs2070016T > C and *FGG* rs1049636T > C. For *FGB* rs1800790G > A and *FGA* rs2070016T > C, this relationship significantly depended on *F13A* Val34Leu and *A2AP* Arg6Trp genotypes. Specifically, in *F13A* 34Val/Val wildtypes, carriage of *FGB* rs1800790A was related to significantly lower fibrinogen levels compared with *FGB* rs1800790GG wildtypes (*p* < 0.01). For *A2AP* 6Arg/Arg wildtypes, a comparable relationship could be found (*p* < 0.04). As these genotype constellations related to lower fibrinogen levels have previously been shown to be associated with reduced inflammatory activity, these findings suggest that the influence of fibrinogen, *F13A* and *A2AP* genotypes on inflammation could affect the control of fibrinogen levels and vice versa.

## 1. Introduction

Expression of fibrinogen depends on gene loci of α-, β- and γ-fibrinogen (*FGA*, *FGB*, *FGG*) and it is increased by acute-phase responses via interleukin 6 (IL6), glucocorticoids and others [1]. Genetic polymorphisms located at these gene loci influence fibrinogen levels [1], with *FGB* rs1800790G > A or a strongly linked genotype being consistently associated with higher fibrinogen levels [2]. An influence of this polymorphism on the transcription rate of the fibrinogen β-chain has been described [3,4]. Different studies confirm the relationship between *FGB* rs1800790G > A and fibrinogen levels in steady-state situations [5] as well as under weak proinflammatory triggers in individuals with unelevated levels of C-reactive protein (CRP) and IL6 [6].

Increasing fibrinogen concentrations are known to result in fibrin clots with increased density and high antifibrinolytic capacity [7]. For this correlation, the factor XIII A-subunit (*F13A*) 34Val/Val wildtype is a necessary precondition, and it is abrogated in carriers of the polymorphism *F13A* 34Leu allele [7]. For full antifibrinolytic capacity of these dense fibrin clot structures, strong incorporation of α_2_-antiplasmin (*A2AP*) is necessary, which is highest in *A2AP* 6Arg/Arg wildtypes [8]. Recently, we described an interaction between fibrinogen, *F13A* and *A2AP* genotypes and inflammation, which could be detected in different cohorts under proinflammatory stimulation [9,10]. Carriage of fibrinogen genotypes known to be related to higher fibrinogen levels at steady-states (e.g., carriage of *FGB* rs1800790G > A minor allele(s)) were associated with reduced inflammatory activity if the presence of the *F13A* 34Val/Val wildtype gave the optimal background for highly crosslinked fibrin gels [7]. Similar results with respect to inflammatory activity could be found for these fibrinogen genotypes when the presence of *A2AP* 6Arg/Arg wildtype predisposed for a high antifibrinolytic capacity [8]. These relationships most likely could be explained with dense fibrin gels, with high antifibrinolytic capacity being more effective in controlling inflammatory processes [11,12].

As the association between fibrinogen genotypes and fibrinogen levels as well as the aforementioned influence of fibrinogen, *F13A* and *A2AP* genetics on inflammation are likely to influence each other, we tested in the present study the following hypothesis:

Fibrinogen genotypes known to be associated with higher fibrinogen levels at steady-state conditions (specifically *FGB* rs1800790G > A as well as polymorphisms of *FGA* and *FGG*) are related to reduced fibrinogen levels under proinflammatory stimulation, when *F13A* 34Val/Val wildtype and/or *A2AP* 6Arg/Arg wildtype is given.

We tested this hypothesis on a cohort of pregnant women, which was extensively characterized for fibrinogen and C-reactive protein (CRP) over the course of pregnancy. Pregnancy is known to be a proinflammatory condition with elevated biomarkers of inflammation [13,14]. Furthermore, pregnancy complications like pre-eclampsia or uteroplacental dysfunction have been described to be associated with increased inflammatory activity [13,14].

## 2. Materials and Methods

### 2.1. Patients

The influence of genotype constellations potentially predisposing for dense fibrin clot structures and inflammatory activity on fibrinogen levels under proinflammatory stimulation was assessed in a cohort of 639 patients (2218 observations), followed during pregnancy because of history of fetal loss (FL), placental dysfunction (PD), pregnancy complications or venous thromboembolism (VTE). CRP and fibrinogen levels were determined regularly with each presentation. Data were analyzed anonymously.

### 2.2. Laboratory Analytics and Parameter Definition

Genotypes of *A2AP* (Arg6Trp) rs2070863 (C > T), *F13A* Val34Leu (rs5985), *FGA* rs6050 (A > G), *FGA* rs2070016 (T > C), *FGA* rs2070006 (G > A), *FGB* −455G > A (rs1800790), *FGB* rs1800788 (C > T), and *FGG* rs1049636 (T > C) as well as CRP levels were characterized as described previously [9,10]. Quantification of fibrinogen levels was performed using Clauss’ method with routine diagnostics (Diagnostica Stago, Asnières sur Seine, France).

### 2.3. Statistical Analysis

For presentation of data distribution frequencies, mean standard deviation (SD), median and interquartile range (IQR) or cumulative probability plots were given, as indicated. For univariate analyses on fibrinogen levels, *t*-tests were performed and *p*-values were calculated. Multivariate analyses of fibrinogen levels were performed by logistic regression analysis adjusting for stage of pregnancy (trimester), age, primary diseases, and ethnicity. If indicated, these analyses were stratified for CRP elevation, *F13A* Val34Leu genotype or *A2AP* Arg6Trp genotype. To evaluate for potential interactive effects, corresponding interaction terms were included in logistic regression models (*p* for homogeneity) [15]. Statistical analyses were performed using Stata Statistical Software 10.1 (StataCorp, College Station, TX, USA).

## 3. Results

### 3.1. Patient Characteristics

The median age of patients included in the study was 32.1 years (IQR, 27.6–36.5), and the following primary diagnoses were reasons for presentation in our hemostasis out-patient unit: history of miscarriage (38%), actual or history of placental dysfunction (21%), history of deep vein thrombosis (DVT) (14%), history of pulmonary embolism (PE) (3.8%). Patients presented regularly during pregnancy as well as 12 weeks postpartum. Until the end of the first, second, and third trimester, 277 (12.5%), 864 (39.0%), and 894 (40.3%) observations were available, respectively. Additionally, 183 (8.3%) observations until 12 weeks postpartum were included in the study.

The distributions of clinical and laboratory data are summarized in Table 1.

### 3.2. Courses of Fibrinogen and CRP Levels during Pregnancy and Postpartum

Mean fibrinogen levels continuously increased with ongoing pregnancy (first trimester, 3.74 ± 0.94 mg/L, second trimester, 4.42 ± 0.96 mg/L, third trimester, 5.20 ± 1.06 mg/L). During the 12 weeks postpartum, a considerable decline in fibrinogen levels could be recognized (3.23 ± 0.81 mg/L). The frequency of CRP elevations ≥10 mg/L increased from 7.94% (first trimester) to 14.0% and 12.1% in the second and third trimesters, respectively. In the interval postpartum, only a small number of patients (2.7%) exhibited elevated CRP levels. For mean CRP levels, a comparable characteristic could be found (first trimester, 5.3 ± 8.12 mg/L, second trimester, 5.84 ± 5.21 mg/L, third trimester, 5.82 ± 7.39 mg/L, postpartum, 4.18 ± 3.53 mg/L).

### 3.3. Fibrinogen Levels in Dependence of Fibrinogen Genotypes with Consideration of CRP

We tested the association of fibrinogen genotypes as well as of *F13A*, *F13B* and *A2AP* with fibrinogen levels. The findings are given in Table 2.

While carriage of the *FGB* rs1800790 A allele exhibited only a very small effect on fibrinogen concentration, carriage of the minor alleles of *FGA* rs2070016T > C or *FGG* rs1049636T > C influenced fibrinogen levels significantly. Interestingly, in our cohort, carriage of the minor alleles of *FGB* rs1800790G > A and *FGA* rs2070016T > C was related to lower fibrinogen levels compared with the corresponding wildtypes, and for *FGG* rs1049636T > C higher fibrinogen levels could be detected. For *F13A* Val34Leu, *F13B* His95Arg and *A2AP* Arg6Trp fibrinogen levels were equal for wildtypes and minor allele carriers.

As expected, CRP elevation itself was very strongly associated with fibrinogen levels in the total study population (CRP <5 mg/L, 4.25 ± 1.06 mg/L, CRP ≥5 mg/L, 5.18 ± 1.14 mg/L, *p* < 0.0001; CRP <10 mg/L, 4.41 ± 1.10 mg/L, CRP ≥10 mg/L, 5.52 ± 1.23 mg/L, *p* < 0.0001). When considering inflammatory activity as measured by CRP elevation, a different behavior of the tested fibrinogen genotypes with respect to fibrinogen levels could be found (Table 3).

While for *FGB* rs1800790G > A in all CRP defined strata a significant association with fibrinogen levels was missing, a strong relationship was found for *FGA* rs2070016T > C in those patients with CRP levels ≥10 mg/L. In this case, minor allele carriers presented with significantly lower fibrinogen levels compared to the wildtypes. For *FGG* rs1049636T > C, minor allele carriers with low inflammatory activity exhibited higher fibrinogen levels than the corresponding wildtypes. With high inflammatory activity (CRP ≥10 mg/L), this genotype–phenotype relation tended to reverse. These findings could be confirmed in multivariate analyses adjusting for trimester, age, disease and ethnicity (data not shown).

### 3.4. Fibrinogen Levels and Dependence of Fibrinogen Genotypes with Consideration of F13A Val34Leu Genotype

As described previously, a strong correlation between fibrinogen level and fibrin clot density depends on the presence of the *F13A* 34Val/Val wildtype [7]. Furthermore, reduced inflammatory activity in *FGB* rs1800790 A-allele carriers could be found only in *F13A* 34Val/Val wildtypes, thus, *F13A* 34Val/Val wildtype seems to be a necessary precondition for this effect [9]. We therefore tested the relationship between fibrinogen genotypes and fibrinogen levels in *F13A* Val34Leu defined strata (Table 4).

Considering *FGB* rs1800790G > A, carriage of the minor allele was associated with significantly lower fibrinogen levels if the necessary background (*F13A* 34Val/Val) was given. In carriers of *F13A* 34Leu this relation vanished. Heterogeneity for this effect between both *F13A* Val34Leu defined strata was statistically significant in univariate as well as in multivariate analyses (*p* for homogeneity: 0.019 and 0.002, respectively). A similar tendency could be found for *FGA* rs2070016T > C. In *F13A* 34Val/Val wildtypes, carriage of the *FGA* rs2070016T > C minor allele was associated with lower fibrinogen levels compared with *FGA* rs2070016T > C wildtypes. In *F13A* 34Leu carriers, this effect of *FGA* rs2070016T > C genotype on fibrinogen levels was considerably diminished. Heterogeneity between both *F13A* Val34Leu strata was not statistically significant. For *FGG* rs1049636T > C, minor allele carriage was robustly associated with higher fibrinogen levels in both *F13A* Val34Leu strata.

We additionally evaluated whether *F13A* Val34Leu or *A2AP* Arg6Trp would have an influence on fibrinogen levels. Neither for *F13A* Val34Leu (fibrinogen levels: 34Val/Val, 4.54 ± 1.12 g/L, 34Leu, 4.53 ± 1.16 g/L, *p* = 0.56) nor for *A2AP* Arg6Trp (fibrinogen levels: 6Arg/Arg, 4.51 ± 1.17 g/L, 6Trp, 4.55 ± 1.15, *p* = 0.21) did fibrinogen levels differ significantly between wildtypes and minor allele carriers.

### 3.5. Fibrinogen Levels in Dependence of Fibrinogen Genotypes with Consideration of A2AP Arg6Trp Genotype

We finally tested whether the *A2AP* Arg6Trp genotype influenced the relationship between fibrinogen genotype and level. This assumption was based on the facts that fibrin clots of *A2AP* 6Arg/Arg wildtypes are known to have a higher antifibrinolytic capacity [8], and that this genotype influences the relationship between fibrinogen genotypes and inflammation [10]. The results of these analyses are given in Table 4. The findings resembled those for *F13A* Val34Leu. In individuals predisposed for a higher antifibrinolytic capacity (*A2AP* 6Arg/Arg wildtypes), carriage of the *FGB* rs1800790 A allele was related to lower fibrinogen levels. The same holds true for *FGA* rs2070016T > C. Here, the differences in fibrinogen levels were especially pronounced, and heterogeneity between both *A2AP* Arg6Trp defined strata was significant (*p* for homogeneity: 0.005). For *FGG* rs1049636T > C, again, carriage of the minor allele was robustly associated with higher fibrinogen levels in both *A2AP* Arg6Trp defined strata.

## 4. Discussion

The genetic background of fibrinogen synthesis is well-described, and different genetic polymorphisms of the fibrinogen gene loci have been shown to influence basal fibrinogen levels [1,16]. The most consistently reported polymorphisms identified to influence fibrinogen levels are located in the promotor region of *FGB*, which has many different regulatory elements [1]. Of them, *FGB* rs1800790G > A is a prominent example [17,18]. Electrophoretic mobility shift assays identified differences in the binding characteristics of various nuclear proteins, with *FGB* fragments carrying *FGB* rs1800790G > AG- and A alleles, respectively [3,4]. Furthermore, an increased basal rate of transcription could be detected for recombinant constructs with *FGB* rs1800790G > AA alleles compared to those with the corresponding G alleles [3,4]. Meanwhile, many different studies confirm increased basal fibrinogen levels in carriers of the *FGB* rs1800790G > A minor allele [5,6]. Regarding the behavior of fibrinogen levels under proinflammatory stimulation, the AIRGENE study added interesting details [6,19]. In this study, air pollution as measured by ambient particulate matter was used as a marker of proinflammatory stimulation, and the relationship between fibrinogen and the 5-day average of ambient particulate matter was analyzed considering the respective fibrinogen genotypes [19]. Of note, this proinflammatory stimulation did not result in recognizable elevations of CRP or IL6 in the study population [6]. This study revealed an up to 8-fold higher increase in fibrinogen levels under proinflammatory stimulation in *FGB* rs1800790G > A minor allele carriers when compared with *FGB* rs1800790G > A wildtypes [19]. Finally, for *FGB* rs4220G > A, which is in strong linkage disequilibrium with *FGB* rs1800790G > A, a significant interaction between inflammation, as measured by interleukin-6 levels and fibrinogen levels, has been described [20].

For *FGA* rs2070016T > C, which has been evaluated in this study in more detail, different studies identified carriage of the minor allele to be associated with higher fibrinogen levels [5,6,21,22,23]. In the case of *FGG* rs1049636T > C, data are more complex. Most investigations found lower fibrinogen levels in carriers of the corresponding minor allele [6,21,23]. However, in some studies, this relationship was found to be heterogenous between men and women, and only detectable in females [23]. In one study, carriage of the *FGG* rs1049636 C allele was associated with higher fibrinogen levels [24]. Moreover, this polymorphism was related to the levels of circulating γ’ fibrinogen, which is a splice polymorphism of fibrinogen with special functionalities [25,26].

Beside these polymorphisms located in fibrinogen gene loci, other loci were reported to be involved in genetic regulation of the strength of fibrinogen synthesis. These loci are involved in the regulation of inflammatory responses, with the loci of interleukin-6 receptor (IL6R) and interferon regulatory factor 1 (IRF1) being two examples [21,22].

Our analyses were performed on a cohort of pregnant women. Pregnancy is known to represent a proinflammatory trigger with elevated biomarkers of acute-phase response [13,14]. This proinflammatory stimulation is increased further in cases of pregnancy complications such as uteroplacental dysfunction [13,14]. Thus, in our view, a cohort of pregnant women is well-suited to test the hypothesis of our study, as a proinflammatory trigger is given. However, it is important to underscore that a generalization of our findings should only made with caution.

The results of the present study are seemingly in opposite to the findings reported earlier on genetic regulation of fibrinogen levels. However, they are in agreement with the hypothesis to be tested. In our population, *FGB* rs1800790 A-allele or *FGA* rs2070016 C-allele carriage was related to lower fibrinogen levels (Table 2). No obvious interaction with inflammatory activity as measured by CRP levels could be found (Table 3). Furthermore, in individuals with a genetic background predisposing for dense fibrin gels with higher antifibrinolytic capacity in situations, when fibrinogen levels are high (i.e., *F13A* 34Val/Val wildtypes and/or *A2AP* 6Arg/Arg wildtypes), *FGB* rs1800790G > A or *FGA* rs2070016T > C minor allele carriers exhibited significantly lower fibrinogen levels (Table 4) compared with the corresponding wildtypes. When comparing the relationship between *FGB* rs1800790G > A or *FGA* rs2070016T > C genotype and fibrinogen level in strata, defined by absence or presence of *F13A* 34Leu, a significant heterogeneity was detectable (Table 4). The same holds true for strata defined by *A2AP* 6Trp carriage (*p* = 0.09) (Table 4).

As reflected by the hypotheses of our study, we think the findings are plausible when considering the interactive effects of fibrinogen, *F13A* and *A2AP* genetics, described previously [9,10,11,27]. Carriage of *FGB* rs1800790 A allele(s) or *FGA* rs2070016 C allele(s) is associated with significantly lower inflammatory activity, if *F13A* 34Val/Val wildtypes and/or *A2AP* 6Arg/Arg wildtypes are considered. This effect can be demonstrated for CRP levels in patients suffering from rheumatoid arthritis (RA) as well as patients with non-autoimmune inflammations (non-RA). In RA patients, clinical disease activity scores (disease activity score of 28 joints) indicate lower inflammatory activity in these constellations, as well [10]. Thus, we assume that a “kinetic” view could help to clarify the putative discrepancies. Individuals carrying *FGB* rs1800790 A allele(s) or *FGA* rs2070016 C allele(s) exhibit higher basal fibrinogen levels [17,18]. In an early phase of proinflammatory stimulation, in these individuals, fibrinogen levels exhibit a steeper increase compared with the corresponding wildtypes [19]. If *F13A* 34Val/Val wildtype and/or *A2AP* 6Arg/Arg wildtype are also given, these situations with higher fibrinogen levels result in the generation of denser fibrin gels [7] with a higher antifibrinolytic capacity [8,28,29,30]. Hereby, a more effective confinement of inflammatory foci is achieved [12,31], which helps to shorten the duration of proinflammatory stimulation. Therefore, after an appropriate period, in individuals with these specific genetic backgrounds, inflammation as well as the trigger for fibrinogen synthesis are sufficiently suppressed to result in lower fibrinogen levels compared with individuals with a different genetic background.

In summary, we could demonstrate that genotype constellations of fibrinogen, *F13A* and *A2AP*, which predispose for reduced inflammatory activity [9,10,11] are related to lower fibrinogen levels in individuals under longstanding proinflammatory stimulation. The differences in fibrinogen levels for the different genotype constellations are rather small. Thus, it is tempting to assume that the findings of our study to have no direct medical implications. However, as previously shown by our group, seemingly small effects of these genotype constellations on CRP levels have a strong impact on a CRP-based predictive algorithm in respect of radiographic progression in spondylarthritis [32,33].

Thus, knowledge on the influence of fibrinogen, *F13A* and *A2AP* genotypes on fibrinogen expression could be of importance for future approaches on diagnostic algorithms based on fibrinogen levels.

## 5. Conclusions

Genetic polymorphisms of *F13A* and *A2AP*, which are known to influence the process of fibrin crosslinking as well as antifibrinolytic capacity of fibrin clots, influence genetic regulation of fibrinogen synthesis by genetic variability of the fibrinogen gene loci *FGB*, *FGA* and *FGG*. It could be assumed that this phenomenon is related to their influence on inflammation control. As our findings have been derived from a cohort of pregnant women, confirmatory studies in other patient groups under proinflammatory stimulation are needed.

## Figures and Tables

**Table 1:** Clinical and laboratory characteristics of the study population.

Parameter	Characteristic
Age [years], median (IQR)	32.1 (27.6–36.5)
Miscarriage	38%
Placental dysfunction	21%
Deep vein thrombosis	14%
Pulmonary embolism	3.8%
Fibrinogen [g/L], mean ± SD	4.54 ± 1.17
CRP [mg/L], mean ± SD	5.54 ± 6.38
*Frequency of minor allele carriers:*	
*FGB* rs1800790G > A	42%
*FGB* rs1800788C > T	38%
*FGA* rs6050A > G	49%
*FGA* rs2070016T > C	34%
*FGA* rs2070006G > A	63%
*FGG* rs1049636T > C	46%
*F13A* Val34Leu	46%
*F13B* His95Arg	18%
*A2AP* Arg6Trp	39%

IQR, interquartile range, CRP, C-reactive protein, SD, standard deviation, *FGB*, β-fibrinogen, *FGA*, α-fibrinogen, *FGG*, γ-fibrinogen, *F13A*, factor XIII A-subunit, *F13B*, factor XIII, B-subunit, *A2AP*, α_2_-antiplasmin.

**Table 2:** Association of genotypes of fibrinogen, *F13A*, *F13B* and *A2AP* polymorphisms with fibrinogen levels.

	Fibrinogen Level [g/L], Mean ± SD		
Genotype	Wildtype	Minor Allele	*p* Value
*FGB* rs1800790G > A	4.56 ± 1.21	4.51 ± 1.11	0.15
*FGB* rs1800788C > T	4.53 ± 1.13	4.51 ± 1.21	0.41
*FGA* rs6050A > G	4.53 ± 1.15	4.54 ± 1.18	0.59
*FGA* rs2070016T > C	4.58 ± 1.21	4.42 ± 1.07	0.002
*FGA* rs2070006G > A	4.51 ± 1.15	4.53 ± 1.17	0.58
*FGG* rs1049636T > C	4.43 ± 1.16	4.63 ± 1.15	0.0002
*F13A* Val34Leu	4.54 ± 1.18	4.53 ± 1.16	0.42
*F13B* His95Arg	4.53 ± 1.15	4.53 ± 1.21	0.51
*A2AP* Arg6Trp	4.51 ± 1.17	4.55 ± 1.15	0.21

SD, standard deviation, *FGB*, β-fibrinogen, *FGA*, α-fibrinogen, *FGG*, γ-fibrinogen, *F13A*, factor XIII A-subunit, *F13B*, factor XIII B-subunit, *A2AP*, α_2_-antiplasmin.

**Table 3:** Association of genotypes of fibrinogen polymorphisms with fibrinogen levels in dependence of inflammatory activity.

	Fibrinogen Level [g/L], Mean ± SD		
Genotype	Wildtype	Minor Allele	*p* Value
***FGB rs1800790G > A***			
CRP < 5 mg/L	4.24 ± 1.10	4.25 ± 1.00	0.56
CRP < 10 mg/L	4.43 ± 1.15	4.40 ± 1.04	0.29
CRP ≥ 10 mg/L	5.53 ± 1.19	5.51 ± 1.30	0.44
***FGA rs2070016T > C***			
CRP < 5 mg/L	4.26 ± 1.11	4.18 ± 0.98	0.08
CRP < 10 mg/L	4.43 ± 1.14	4.34 ± 1.04	<0.05
CRP ≥ 10 mg/L	5.62 ± 1.20	5.21 ± 1.01	0.01
***FGG rs1049636T > C***			
CRP < 5 mg/L	4.13 ± 1.03	4.35 ± 1.08	0.0001
CRP < 10 mg/L	4.31 ± 1.08	4.51 ± 1.12	0.0002
CRP ≥ 10 mg/L	5.64 ± 1.26	5.39 ± 1.08	0.07

SD, standard deviation, *FGB*, β-fibrinogen, *FGA*, α-fibrinogen, *FGG*, γ-fibrinogen, CRP, C-reactive protein.

**Table 4:** Association between fibrinogen genotypes and fibrinogen levels in dependence of *F13A* Val34Leu and *A2AP* Arg6Trp genotypes.

	Fibrinogen Level [g/L], Mean ± SD		
Genotype	Wildtype	Minor Allele	*p* Value
***FGB rs1800790G > A***			
*F13A* 34Val/Val	4.62 ± 1.23	4.45 ± 1.12	<0.01 *
*F13A* 34Leu	4.50 ± 1.18	4.58 ± 1.11	0.15 *
*A2AP* 6Arg/Arg	4.56 ± 1.25	4.44 ± 1.07	<0.04 ^#^
*A2AP* 6Trp	4.52 ± 1.14	4.59 ± 1.17	0.22 ^#^
***FGA rs2070016T > C***			
*F13A* 34Val/Val	4.60 ± 1.26	4.38 ± 1.04	<0.004
*F13A* 34Leu	4.56 ± 1.16	4.46 ± 1.10	0.1
*A2AP* 6Arg/Arg	4.61 ± 1.24	4.33 ± 1.03	<0.0001 ^$^
*A2AP* 6Trp	4.54 ± 1.17	4.59 ± 1.12	0.28 ^$^
***FGG rs1049636T > C***			
*F13A* 34Val/Val	4.44 ± 1.19	4.63 ± 1.19	0.006
*F13A* 34Leu	4.43 ± 1.14	4.62 ± 1.13	0.006
*A2AP* 6Arg/Arg	4.41 ± 1.17	4.61 ± 1.16	<0.002
*A2AP* 6Trp	4.47 ± 1.15	4.65 ± 1.15	0.02

SD, standard deviation, *FGB*, β-fibrinogen, *FGA*, α-fibrinogen, *FGG*, γ-fibrinogen, *F13A*, factor XIII A-subunit, *A2AP*, α_2_-antiplasmin.* *p* for homogeneity (*F13A* 34 al/Val vs. *F13A* 34Leu): 0.019. ^#^ *p* for homogeneity (*A2AP* 6Arg/Arg vs. *A2AP* 6Trp): 0.09. ^$^ *p* for homogeneity (*A2AP* 6Arg/Arg vs. *A2AP* 6Trp): 0.005.

## Data Availability

Data are available upon reasonable request.
